# IgG4-related hypophysitis diagnosed by retroperitoneal mass biopsy in a patient presenting with abducens nerve palsy: A case report (CARE-compliant article)

**DOI:** 10.1097/MD.0000000000022484

**Published:** 2020-10-02

**Authors:** Takeshi Imai, Souichirou Shibata, Kensuke Shinohara, Kenzo Sakurai, Masahiro Horiuchi, Kensuke Sakai, Shiko Asai, Yasuhiro Hasegawa

**Affiliations:** a Department of Neurology; b Tama Medical Practice Center; c Clinical training center; d Department of Metabolism and Endocrinology, Kawasaki Municipal Tama Hospital, Kawasaki, Kanagawa, Japan; e Department of Internal Medicine, Division of Neurology, St Marianna University School of Medicine, Kawasaki, Kanagawa, Japan.

**Keywords:** hypophysitis, immunoglobulin G4, abducens nerve palsy, retroperitoneal fibrosis

## Abstract

**Rationale:**

Immunoglobulin G4 (IgG4)-related hypophysitis is a rare disorder which often requires invasive pituitary gland biopsy to confirm its diagnosis. We present a case whereby peripheral organ lesion biopsy and imaging findings were sufficient for the diagnosis.

**Patient concerns:**

A 77-year-old man with diplopia was referred to our department by an opthomologist who had diagnosed the patient with right abducens nerve palsy.

**Diagnoses:**

Head magnetic resonance imaging revealed enlargement of the pituitary gland and pituitary stalk, while hormonal analysis revealed panhypopituitarism, thereby indicating a diagnosis of hypophysitis. Abdominal computed tomography imaging revealed a solid mass that encompassed the left kidney ureter. Although the patient did not have an increase in serum IgG4, a biopsy of the periureteral mass revealed infiltrating plasma cells that were positive when stained for IgG4.

**Interventions:**

The patient was given corticosteroid pulse therapy (methylprednisolone: 1 g × 3 days), followed by oral corticosteroids (prednisolone, 0.5 mg/kg/d).

**Outcomes:**

The right abducens nerve palsy improved and the pituitary lesion shrank after the initiation of corticosteroid treatment.

**Conclusion:**

Based on the diagnosis of IgG4-related disease in the retroperitoneal organ and response to corticosteroid treatment, this patient was diagnosed with IgG4-related hypophysitis. This hypophysitis caused enlargement of the pituitary gland with resulting nerve compression, causing abducens nerve palsy. When IgG4-related hypophysitis is suspected, a thorough examination of other organ lesions and biopsy should be considered.

## 1 Introduction

Immunoglobulin G4 (IgG4)-related diseases are systemic and chronic inflammatory diseases characterized by elevated concentrations of serum IgG4 and a marked increase in the infiltration by IgG4-positive plasma cells into the tissues of lesions.[^1^,^2^] The pituitary gland and the surrounding tissue can often become the site of lesions, and such lesions have been used as an explanation for hypophysitis, a rare disorder of unknown etiology. During the 2000s, emerging evidence pointed to an autoimmune origin of certain types of hypophysitis, and in 2009, IgG4-related hypophysitis was proposed as a new disease.^[3]^ In this report, we present a case of a patient who presented with abducens nerve palsy which we assessed to be caused by IgG4-related hypophysitis.

## 2 Case report

Starting a month before admission, a 77-year-old man described feeling fatigued and experiencing double vision. His symptoms gradually worsened, leading him to consult an ophthalmologist who diagnosed him with right abducens nerve palsy. The patient was subsequently referred to our department and was hospitalized in order to perform detailed investigations.

Findings on admission: height 165 cm, body weight 56 kg, body temperature 36.2°C, blood pressure 135/85 mm Hg, and peripheral capillary oxygen saturation 98% (room air). His superficial lymph nodes were not palpable, and there was no rash or mucosal eruption. He was lucid and fully conscious, with no sign of disorientation. His corrected visual acuity was 1.0 and 1.2 in the right and left eyes, respectively, and no abnormalities were observed in his anterior segment or fundus. Both pupils were of equal size and round, and demonstrated rapid light reflexes. The patient showed right esotropia, and right abduction disorder was identified in his eye movements; however, the movement did not exceed the median. His other cranial nerves did not show any abnormal findings, and he had no muscle weakness or sensory disturbance.

Laboratory data revealed a white blood cell count of 9.700 cells/μL and a C-reactive protein concentration of 2.43 mg/dL, indicating a mild inflammatory response. There were no notable findings in the biochemistry and coagulation tests. The cerebrospinal fluid was clear, with a cell count of 48 cells/μL (68% mononuclear cells, 32% polymorphonuclear cells), glucose concentration of 84 mg/dL (124 mg/dL blood glucose), protein concentration of 182 mg/dL, and negative oligoclonal bands. Hormonal analysis revealed low levels of free triiodothyronine and free thyroxine, no elevation of thyroid stimulating hormone (TSH), total urinary free cortisol excretion below the level of detection, and low serum cortisol, growth hormone (GH), luteinizing hormone (LH), follicle-stimulating hormone (FSH), and testosterone levels (Table 1). Based on the hormone findings, panhypopituitarism was suspected. His serum immunoglobulin concentrations were 1940 mg/dL, 432 mg/dL, and 137 mg/dL, for IgG, IgA, and IgM, respectively; his soluble IL-2R was 484 U/mL, and angiotensin-converting enzymes was 2.8 IU/L. Antinuclear antibodies, myeloperoxidase antineutrophil cytoplasmic antibodies, proteinase 3 antineutrophil cytoplasmic antibodies, anti-double-stranded-DNA, anti-single-stranded-A, and anti-single-stranded-B antibodies were all negative. Viral antibody tests for herpes simplex virus, varicella zoster virus, cytomegalovirus, human T-cell leukemia virus-1, and human immunodeficiency virus were negative, indicating an absence of infection.

**Table 1:**
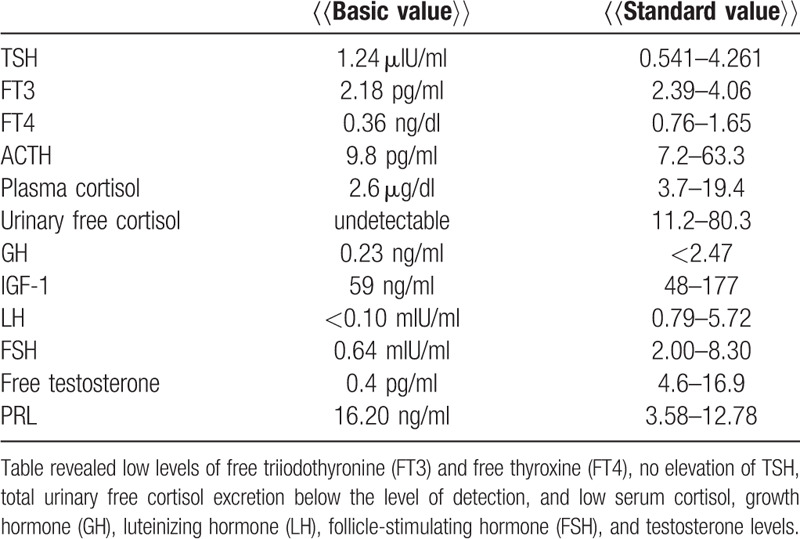
Hormonal analysis.

**Figure 1:**
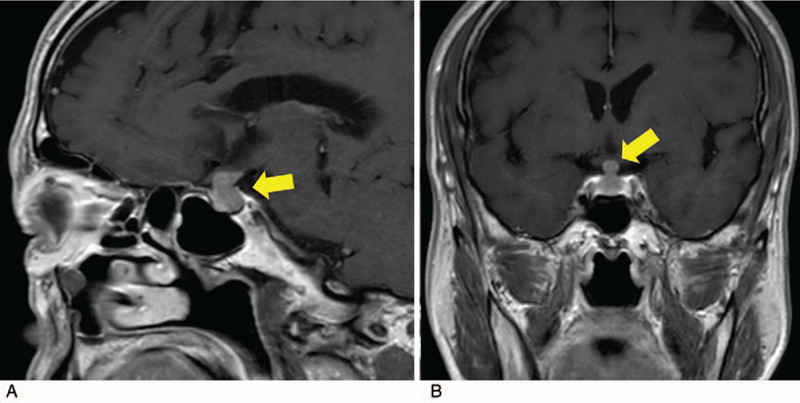
Gadolinium-enhanced T1-weighted brain magnetic resonance images obtained on admission (A: sagittal image, B: coronal image). The images reveal a thickened pituitary stalk and a hyperintense area in the enlarged pituitary gland (arrows).

**Figure 2:**
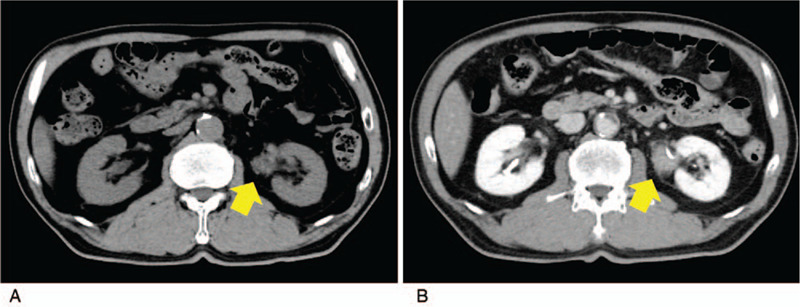
Abdominal computed tomography images on admission. (A: image without contrast enhancement; B: image with contrast enhancement). The images reveal left renal pelvis thickening of the retroperitoneum with encasement of the ureters (arrows).

Contrast-enhanced magnetic resonance imaging (MRI) of the head revealed enlargement of the pituitary gland and pituitary stalk (Fig. 1). Abdominal computed tomography (CT) also showed a mass surrounding the left ureter (Fig. 2).

Load tests were conducted using four hypothalamic hormones: LH-releasing hormone (100 μg), GH-releasing hormone (100 μg), corticotropin-releasing hormone (CRH, 100 μg), and thyrotropin-releasing hormone (TRH, 250 μg) in order to confirm the diagnosis of panhypopituitarism. During the load tests, TSH and adrenocorticotropic hormone levels were maintained in response to stimulation with TRH and CRH, respectively. In contrast, the response to LH and FSH was low, and there was no increase in GH. The patient's free cortisol levels remained low and did not respond to CRH administration. Based on these findings, he was diagnosed with panhypopituitarism, which, based on the imaging findings of swelling spanning from the pituitary gland to the pituitary stalk, had occurred as a result of hypophysitis.

**Figure 3:**
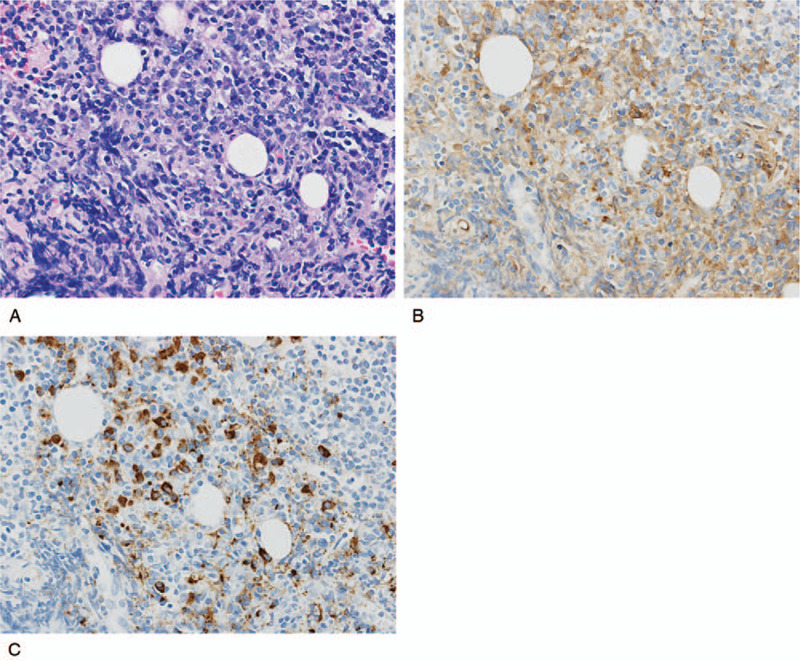
Histological findings of the retroperitoneal mass lesion (×400). (A: hematoxylin and eosin; B: IgG-immunostaining; C: IgG4-immunostaining). IgG immunohistochemical staining revealed that most of the infiltrating plasma cells were positive. IgG4 immunohistochemical staining revealed that more than 50% of the IgG-positive plasma cells were positive for IgG4. IgG = immunoglobulin G.

Additionally, since the abdominal CT revealed possible retroperitoneal fibrosis, the prospect of an IgG4-related disease was considered. Thus, a CT-guided needle biopsy was performed of the solid mass surrounding the left ureter. Histological examination revealed lymphocytic infiltration, which included lymphoplasmacytes. Further IgG4 immunostaining confirmed an abundant infiltration of IgG4-positive plasma cells. The IgG4/IgG ratio was 66.9% and the number of IgG4 positive cells was 175/high power field (Fig. 3). Although serum IgG4 was 129 mg/dL, the patient was diagnosed with IgG4-related retroperitoneal fibrosis based on the pathological findings.

**Figure 4:**
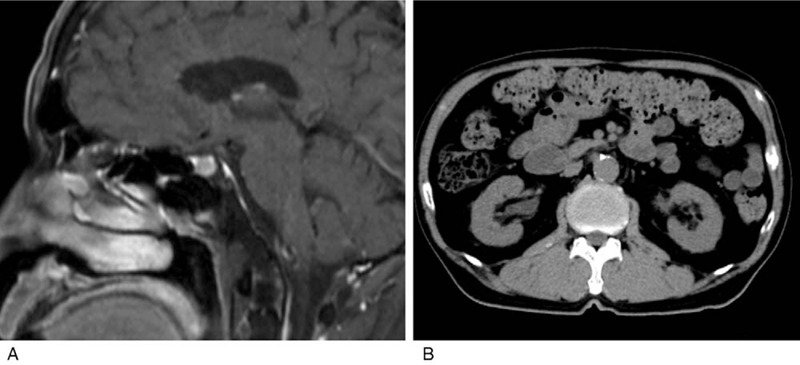
Follow-up imaging 2 months after the diagnosis of IgG4-related disease. A: Magnetic resonance imaging showed improvement of the pituitary lesion. B: Computed tomography showed improvement of the retroperitoneal lesion.

He was started on levothyroxine sodium (12.5 μg/d) for his hypothyroidism, and was given corticosteroid pulse therapy (methylprednisolone: 1 g × 3 days) because, as hypophysitis had developed, his abducens nerve palsy was also considered to be an IgG4-related disease. In addition, oral corticosteroids (prednisolone, 0.5 mg/kg/d) were administered post-therapy, beginning on Day 12 of his admission. He responded well and his right abducens palsy improved. He was discharged on Day 24. Follow-up imaging on Day 35 revealed shrinkage of the pituitary lesions and the retroperitoneal mass (Fig. 4).

## 3 Discussion

IgG4-related hypophysitis has been reported to account for 30% of all cases of hypophysitis.^[4]^ To the best of our knowledge, there have been only 2 similar case reports of patients with an IgG4-related hypophysitis presenting with abducens nerve palsy[^5^,^6^] (Table 2). However, this is the first report of a patient diagnosed with IgG4-related hypophysitis based on a retroperitoneal tissue biopsy.

**Table 2:**
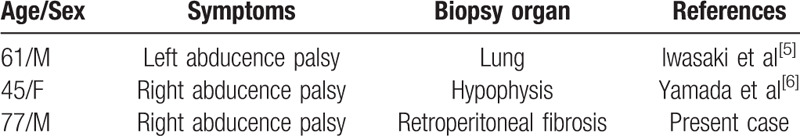
Two similar case reports that describe a patient with an IgG4-related hypophysitis presenting with abducens nerve palsy.

Although the pathological diagnosis of pituitary lesions by biopsy and surgery is indispensable for the definitive diagnosis of IgG4-related hypophysitis, it is not common to perform invasive pituitary biopsies for diagnostic purposes. Consequently, it is generally difficult to obtain a pathological diagnosis.^[7]^ Since IgG4-related hypophysitis is often associated with the involvement of multiple organs, Peripheral organ biopsy may be useful for diagnosing IgG4-related hypophysitis without the need for pituitary biopsies. Leporati et al,^[8]^ proposed the following diagnostic criteria for IgG4-related hypophysitis:

(1) pituitary histology with mononuclear infiltrates (lymphocytes and plasma cells, IgG4 positive plasma cells >10 cells/high-power field);
(2) MRI findings with pituitary enlargement;
(3) biopsy-proven involvement in other organs;
(4) serologic findings of elevated serum IgG4 (>140 mg/dL); and
(5) tumor or symptom response to corticosteroids.

It has been proposed that (1) alone, (2) + (3), or (2) + (4) + (5), can be considered sufficient to make a diagnosis of IgG4-related hypophysitis (Table 3). A guideline of diagnostic criteria for IgG4-related hypophysitis has also been developed in Japan.^[9]^

**Table 3:**
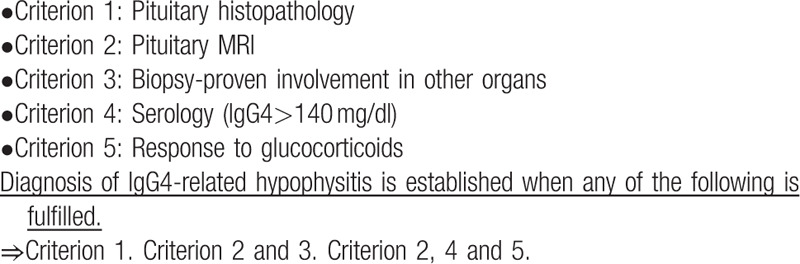
Diagnostic criteria for IgG4-related hypophysitis.

Hypophysitis can be diagnosed if the basal-blood concentration of at least 1 anterior pituitary gland hormone is decreased and its target hormone is likewise decreased, a decreased responsiveness in the anterior pituitary gland hormone secretory stimulation test is seen, diffuse swelling of the pituitary gland, or, hyperplasia of the pituitary stalk are observed in imaging tests, and IgG4-positive plasma cell infiltration is observed in the tissue of lesions present in other organs. In other words, as was the case with our patient, even without confirming the pathology in the pituitary gland or observing elevated serum IgG4, it was possible to diagnose IgG4-related hypophysitis based on pituitary enlargement observed through imaging, and the histology of the retroperitoneal lesion.

Five nerves run across the cavernous sinus, the oculomotor, trochlear, the first and second branches of the trigeminal nerve, the ophthalmic and maxillary nerves, and the abducens nerve. Of these, the abducens nerve is located closest to the pituitary gland. As a result, the abducens nerve was the only cranial nerve affected. It was affected as a result of lateral compression which resulted from the hypophysitis and pituitary enlargement.

The treatment of IgG4-related hypophysitis similar to the of treatment of other IgG4-related diseases. The recommended initial dose of corticosteroids for IgG4-related diseases is 0.5-0.6 mg/kg/d of prednisolone; however, corticosteroid pulses are considered depending on the disease state.^[10]^ In this patient, the hypophysitis was caused by pituitary swelling, and corticosteroid pulse therapy was chosen as the primary treatment. This patient had a rare manifestation of IgG4-related hypophysitis, as it presented with the complication of abducens nerve palsy. The diagnosis was determined based on the finding of IgG4-related disease in the retroperitoneal organ and the patient's responsiveness to corticosteroid treatment.

## 4 Conclusion

The possibility of IgG4 related disease should be considered when findings of hypophysitis are observed in head MRIs of patients presenting with external ophthalmoplegia, and assessment of peripheral organ lesions should be performed.

## Author contributions

**Conceptualization:** Takeshi Imai. Yasuhiro Hasegawa.

**Supervision:** Yasuhiro Hasegawa.

**Visualization:** Takeshi Imai.

**Writing – original draft:** Takeshi Imai.

**Writing – review & editing:** Takeshi Imai. Yasuhiro Hasegawa.
